# Burden of diseases attributable to excess body weight in the Middle East and North Africa region, 1990–2019

**DOI:** 10.1038/s41598-023-46702-y

**Published:** 2023-11-20

**Authors:** Seyed Aria Nejadghaderi, Jessica A. Grieger, Nahid Karamzad, Ali-Asghar Kolahi, Mark J. M. Sullman, Saeid Safiri, Siamak Sabour

**Affiliations:** 1 Safety Promotion and Injury Prevention Research Centre, Department of Epidemiology, School of Public Health and Safety, Shahid Beheshti University of Medical Sciences, Tehran, Iran; 2 School of Medicine, Shahid Beheshti University of Medical Sciences, Tehran, Iran; 3 Adelaide Medical School, University of Adelaide, Adelaide, SA Australia; 4 Robinson Research Institute, University of Adelaide, Adelaide, SA Australia; 5 Department of Persian Medicine, School of Traditional Medicine, Tabriz University of Medical Sciences, Tabriz, Iran; 6 Nutrition Research Center, Tabriz University of Medical Sciences, Tabriz, Iran; 7 Social Determinants of Health Research Center, Shahid Beheshti University of Medical Sciences, Tehran, Iran; 8 Department of Life and Health Sciences, University of Nicosia, Nicosia, Cyprus; 9 Department of Social Sciences, University of Nicosia, Nicosia, Cyprus; 10 Social Determinants of Health Research Center, Department of Community Medicine, Faculty of Medicine, Tabriz University of Medical Sciences, Tabriz, Iran; 11 Clinical Research Development Unit of Tabriz Valiasr Hospital, Tabriz University of Medical Sciences, Tabriz, Iran

**Keywords:** Risk factors, Endocrine system and metabolic diseases, Epidemiology, Obesity

## Abstract

High body mass index (BMI), or excess body weight (EBW), represents a significant risk factor for a range of diseases, including cardiovascular diseases and cancers. The study sought to determine the burden of diseases attributable to EBW in the Middle East and North Africa (MENA) region from 1990 and 2019. The analysis also included an exploration of this burden by age, sex, underlying cause, and sociodemographic index (SDI). We utilized publicly available data from the Global Burden of Disease (GBD) study 2019 to identify the deaths and disability-adjusted life-years (DALYs) of diseases associated with EBW in MENA, spanning the period from 1990 to 2019. The GBD estimated the mean BMI and the prevalence of EBW using hierarchical mixed-effects regression, followed by spatiotemporal Gaussian process regression to determine the most accurate BMI distribution through comparison with actual data. In 2019, there were an estimated 538.4 thousand deaths (95% UI 369.9–712.3) and 17.9 million DALYs (12.9–23.1) attributable to EBW in the region. The DALYs attributable to EBW were higher in men (9.3 million [6.5–12.4]) than in women (8.5 million [6.4–10.8]). The age-standardized death and DALY rates for the diseases associated with EBW increased by 5.1% (− 9.0–25.9) and 8.3% (− 6.5–28.8), respectively, during the study period which was not significant. Egypt had the highest age-standardized mortality rate due to EBW (217.7 [140.0, 307.8]), while Yemen (88.6 [45.9, 143.5]) had the lowest. In 2019, the highest number of DALYs occurred among individuals aged 60 to 64 years old. Furthermore, we found a positive association between a nation's SDI and the age-standardized DALY rate linked to EBW. Cardiovascular disease emerged as the leading contributor to the EBW burden in MENA. The disease burden attributable to EBW showed a non-significant increase in MENA from 1990 to 2019.

## Introduction

Overweight and obesity, collectively named excess body weight (EBW) or having a body mass index (BMI) ≥ 25 kg/m^2^, has shown clear associations with several chronic conditions, including cardiovascular, respiratory, neurological, gastrointestinal, and endocrinological diseases^1,2^. The relationships that EBW has with the burden of cancers^3–5^, osteoarthritis^6^, and other non-communicable diseases^7^ have also been established. EBW is a multifactorial and complex disease associated with multiple biological, psychological and socio-environmental risk factors^8^. It is influenced not only by genetic and epigenetic factors, such as genes of the leptin-melanocortin signaling pathway and the *peroxisome proliferator-activated receptor gamma* gene, but also by environmental factors, drugs, emotional factors, and even gut microbiota dysbiosis^9,10^. EBW has been declared a pandemic that demands global efforts for its prevention^11^.

During the period from 1975 to 2016, there was a notable rise in the age-standardized prevalence of EBW in across almost all regions of the world^12^. In 2017, the age-standardized rates of death and disability-adjusted life years (DALYs) linked to EBW were 109.5 and 3256.0 per 100,000 population, respectively^1^. In 2017, globally, cardiovascular diseases were the largest contributors to the burden of diseases linked to EBW, followed by diabetes and kidney diseases^1^. Moreover, the global age-standardized DALY rate associated with EBW-related diseases increased with advancing age in 2017, exception of for a decrease observed in the 75–84 years age group^1^. Furthermore, globally, the age-standardized DALY rate was higher for males up to 69 years, and then it was higher among females^1^. There was also an inverted-U shaped association between the burden of EBW-attributable diseases and socioeconomic development at regional levels^1^. In 2015, EBW accounted for 10.0% of all deaths and 6.3% of all DALYs in the Eastern Mediterranean Region^13^. Moreover, in 2017, the burden attributable to EBW was most pronounced in the 95 + age group and in regions characterized by intermediate levels of socioeconomic development^1^. The burden attributable to EBW in 2017 was also reported at the global level^1^, and in some regions and countries, such as the Eastern Mediterranean Region^13^ and Colombia^14^.

Economic modeling provides insights into the escalating economic cost of EBW, which is projected to rise from 1.8% of the gross domestic product in 2019 to  an estimated 3.6% by the year 2060^15^. In the case of children aged 2–5 years, a 0.13 decrease in BMI Z score can potentially save more than 36 thousand health-adjusted life years and approximately $31 million in healthcare costs^16^.

A previous study documented the worldwide burden of diseases associated with high BMI in 2017^1^. However, here has not been any previous research on the burden of diseases associated with EBW in the Middle East and North Africa (MENA) region. The MENA region encompasses nations that have substantial variations in the level of social development, and the region has witnessed large changes in social, economic, and cultural patterns. Given the large changes in the dietary behaviors and lifestyles among the region’s population, it is important to evaluate the disease burden attributable to EBW^17^. Therefore, this study assessed the burden of diseases attributable to EBW by age, sex, underlying cause, and socio-demographic index (SDI) in the 21 countries of the MENA region from 1990 to 2019.

## Methods

### Overview

The Global Burden of Disease (GBD), an initiative led by the Institute for Health Metrics and Evaluation (IHME), is an ongoing project aimed at assessing the global disease burden. The most recent edition, GBD 2019, sought to gauge the burden of 369 diseases and injuries along with 87 risk factors for the years 1990 to 2019 across 204 countries and 21 regions^18,19^. Among these regions, the MENA region consists of the following 21 countries: Afghanistan, Algeria, Bahrain, Egypt, Iran, Iraq, Jordan, Kuwait, Lebanon, Libya, Morocco, Oman, Palestine, Qatar, Saudi Arabia, Sudan, the Syrian Arab Republic, Tunisia, Turkey, the United Arab Emirates and Yemen. The Comparative Risk Assessment (CRA) method was employed to model the burden attributed to 87 risk factors, including EBW. The methods used to estimate the disease and injury burdens, as well as the influence of risk factors have been extensively documented in previous publications^18,19^. The fatal and non-fatal data can be obtained from the following websites: https://vizhub.healthdata.org/gbd-compare/ and http://ghdx.healthdata.org/gbd-results-tool. In this study, data on EBW, defined by BMI, were collected from sources such as surveys and systematic literature reviews. Different models were then used to estimate the burden of diseases associated with EBW in each country by age, sex, and SDI.

### Case definition and data sources

BMI was used to determine whether adults were overweight (BMI 25–30.0 kg/m^2^) or obese (BMI > 30 kg/m^2^). The burden of diseases linked to EBW was determined using the GBD 2019 methodology^19^. The prevalence of high BMI was estimated in the GBD 2019, for both children and adults, in 204 countries and territories from 1990 to 2019. The exact methodology used to estimate mean BMI has been reported previously^19,20^. To identify studies that estimated the prevalence of EBW and mean BMI at the national or subnational level, the IHME conducted a comprehensive search of the online Medline database in 2015. This comprehensive literature review was updated using the same process for articles published in 2016 (January 1 to December 31). In addition, a search of the Global Health Data Exchange (GHDx) database was conducted to identify individual-level information from large international or national surveys^19,21^. The search strategy and data sources obtained for each country and territory have been previously published^19,21^.

The IHME extracted information from the literature on sample size, mean BMI, prevalence of overweight, prevalence of obesity, uncertainty measures for overweight and obesity, and locations and years for the most accurate age and sex categories available. In addition, IHME also collected details about each data source such as primary sampling unit, strata, and survey weights, which were used to formulate individual-level microdata and provide more accurate estimates of uncertainty. Three study-level variables were created to provide information on whether: (1) the sample was representative of the population; (2) the study was conducted primarily in urban areas, rural areas, or both; and (3) height and weight data were self-reported or measured. For countries with multiple data sources, all were incorporated into the analyses^19^.

### Estimations of BMI and the prevalence of overweight and obesity

To calculate the avarage BMI, a stratified hierarchical mixed-effects regression was applied to examine the connection between BMI, overweight and obesity in data sources that provided information on all three variables. For this purpose, sex-specific MR-BRT models were used to analyze both overweight and obesity. These models focused on the logit disparity between measured and self-reported data. The coefficients derived from this regression were then used in a spatiotemporal Gaussian process regression (ST-GPR) to model the prevalence of individuals with overweight and obesity in each country by age, sex and year. Multiple distributions were evaluated against the actual data to determine the BMI distribution that most closely resembled it. The final form of the beta distribution was determined using the mean BMI and the prevalence of being classified as overweight or obese in each country, by age, sex and year. For a more comprehensive understanding of the modeling process please refer to the GBD capstone paper^19^.

The prevalence of overweight and obesity was calculated via ST-GPR models^19,22^. To enhance estimates in countries with limited data, three country-level covariates were used: per capita energy intake with a 10-year lag, country latitude, and the urban population proportion. The selection of these three variables was informed by extensive prior research^20,22^, as they demonstrated the most favorable fit and coefficients that aligned with anticipated trends^19,21^.

In GBD 2019, Meta-Regression with Bayesian priors, Regularization and Trimming (MR-BRT) was used to adjust for self-report bias. Sex-specific MR-BRT models were conducted, with a fixed effect for super-region on the logit difference between overweight and obesity, depending on whether they were measured or self-reported^19^.

### Data on the estimated relative risk

The evidence substantiating the relationships high BMI had with the different diseases was evaluated using the CRA approach. In brief, the CRA contains six main steps, which include: identification of the risk-outcome pairs to utilise in the analysis; estimating the relative risk as a function of exposure; estimating exposure levels and distributions; determining the counterfactual level of exposure; computing the fraction of the population that can be attributed and the burden that can be attributed; and estimating the mediation of various risk factors^19^. Additional information on this estimation process has been published previously^19^. The evidence indicated that the following conditions are associated with high BMI: cardiovascular diseases, diabetes and kidney diseases, neoplasms, gallbladder and biliary diseases, asthma, cataracts, osteoarthritis, low back pain, gout, as well as Alzheimer’s disease and other dementias^19^.

### Estimation of the proportion of diseases associated with EBW

The population-attributable fraction (PAF) was employed to gauge the disease burden linked to EBW by country, age group, sex, and year. The theoretical minimum risk exposure level (TMREL), measured using the estimated relative risk (RR), was used in GBD 2019 to estimate the level of exposure to each risk factor that would minimize the chances of suffering any EBW-related burden. The following formula was used to calculate the PAF:

$$PAF = \frac{{\mathop \smallint \nolimits_{i = n}^{m} RR\left( x \right)P\left( x \right)d\left( x \right) - RR\left( {TMREL} \right)}}{{\mathop \smallint \nolimits_{i = n}^{m} {\text{RR}}\left( {\text{x}} \right){\text{P}}\left( {\text{x}} \right){\text{d}}\left( {\text{x}} \right)}}$$

n is the lowest level of exposure observed, m is the highest level of exposure recorded, RR(x) is the relative risk at an exposure level of x and P(x) is the fraction of risk exposure^19^.

### Compilation of results

In GBD 2019, the number of deaths and DALYs linked to EBW were estimated for each nation, age group, sex, year and attributable disease by multiplying the appropriate numbers with the corresponding PAFs. Detailed information regarding the methods utilized for estimating the number of deaths and the disease-attributable DALYs has been reported previously^19^. All estimations were presented as numerical counts, proportions (PAFs), and age- standardized rates per 100,000. Furthermore, they were accompanied by 95% uncertainty intervals (UIs) which encompassed the 25th and 975th values of the 1000 ordered draws^19^. The association between socio-economic development and the diseases attributable to EBW was also examined. The GBD project measures socio-economic development using SDI, which is a composite measure comprised of three components: the fertility rate for women under 25 years old, the average years at school for individuals over 15 years old, and the lag-distributed income per capita. SDI ranges from the least developed (0) to the most developed^1^.

### Ethical approval

The present study was approved by the ethics committee of Shahid Beheshti University of Medical Sciences (IR.SBMU.PHNS.REC.1401.101). All methods were performed in accordance with the national guidelines and regulations. This study is based on publicly available data and solely reflects the opinion of its authors and not that of the Institute for Health Metrics and Evaluation.

## Results

### The Middle East and North Africa region

In 2019, there were 538.4 thousand deaths (95% UI 369.9 to 712.3) linked to EBW in the MENA region. This accounted for 17.4% (12.2 to 22.5) of all deaths in the region (Table 1). Also in 2019, there were 272.3 (177.8 to 368.5) thousand deaths in men that were attributable to EBW and 266.2 (190.4 to 344.0) thousand deaths among women (Table S1). The age- standardized death rate in 2019 (133.6 [90.0 to 179.0] per 100,000) was 5.1% higher (-9.0 to 25.9) than in 1990 (127.1 [79.2 to 181.3]) (Table S2). In 2019, EBW caused 17.9 million DALYs (12.9 to 23.1), which accounted for 10.9% (7.9 to 13.9) of all DALYs in both males and females combined, with 9.3 (6.5 to 12.4) million DALYs in men and 8.5 (6.4 to 10.8) million DALYs among females (Table S3). Over the period 1990-2019, the age-standardized DALY rate (per 100,000) increased from 3488.8 (2262.5 to 4798.5) to 3777.2 (2692.6 to 4943.3), a relative increase of 8.3% (− 6.5 to 28.8) (Table S4).

**Table 1:** The burden of diseases attributable to excess body weight in the Middle East and North Africa region in 2019 and the percentage change in age-standardized rates over the period 1990–2019.

	Deaths (95% UI)				DALY (95% UI)			
	Counts (2019)	PAF (2019)	ASRs (2019)	% change in ASRs 1990–2019	Counts (2019)	PAF (2019)	ASRs (2019)	% change in ASRs1990-2019
North Africa and Middle East	538,448 (369,917, 712,329)	17.4 (12.2, 22.5)	133.6 (90, 179)	5.1 (− 9, 25.9)	17,887,734 (12,867,706, 23,131,993)	10.9 (7.9, 13.9)	3777.2 (2692.6, 4943.3)	8.3 (− 6.5, 28.8)
Afghanistan	22,048 (13,613, 32,635)	8.8 (5.7, 12)	177.3 (109.1, 256.1)	33.6 (− 1.9, 103.3)	791,434 (500,585, 1,153,716)	4.6 (3, 6.5)	5098.6 (3230.9, 7244.7)	29.9 (− 3.7, 93.4)
Algeria	36,087 (23,393, 50,726)	17.9 (12.2, 24.1)	125.2 (80.3, 177.3)	− 5 (− 27, 32.4)	1,177,016 (829,801, 1,579,462)	11.6 (8.3, 14.9)	3339.2 (2312.7, 4478.5)	− 4.1 (− 26.1, 28.9)
Bahrain	1127 (768, 1504)	26.4 (19.3, 32.4)	161.7 (107.1, 219.5)	− 23.6 (− 37.9, − 2.5)	48,979 (35,007, 63,284)	17.2 (12.9, 20.9)	4297.4 (2997.1, 5612.6)	− 21.2 (− 35, − 3.1)
Egypt	130,342 (83,824, 184,741)	23.2 (16, 30)	217.7 (140, 307.8)	25.3 (− 3.4, 64.9)	4,217,365 (2,842,298, 5,775,492)	16 (11.3, 20.3)	5929.6 (3973.1, 8107.8)	27.6 (0.4, 65.3)
Iran	61,415 (43,342, 81,358)	15.7 (11.1, 20.7)	91.7 (63.9, 122.1)	2.8 (− 13.1, 32.6)	1,989,457 (1,441,100, 2,561,406)	10.1 (7.3, 13)	2580.9 (1845.1, 3337.3)	6.7 (− 9.5, 35.6)
Iraq	36,481 (23,496, 51,146)	20.3 (13.7, 26.3)	172 (111.2, 237.2)	− 11.5 (− 30.5, 11)	1,236,797 (841,166, 1,672,506)	12 (8.4, 15.5)	4793.1 (3232.7, 6505.3)	− 13.1 (− 30.6, 9.3)
Jordan	7548 (5303, 9857)	23.4 (16.9, 29)	137.1 (94.3, 183.4)	− 23.1 (− 36.3, − 2.4)	262,618 (190,314, 340,017)	12.3 (9.1, 15.1)	3701.6 (2637, 4819.4)	− 19.5 (− 32.3, − 1)
Kuwait	2317 (1596, 3014)	23.1 (16.8, 29)	93.6 (62.4, 125.4)	− 23.4 (− 34.9, − 7.4)	108,657 (80,242, 137,859)	14.3 (11, 17.3)	3156.1 (2248.5, 4046.2)	− 11.9 (− 23.1, 2.5)
Lebanon	6165 (3951, 8493)	18.2 (11.7, 24.6)	120.6 (77.6, 166.4)	− 5.9 (− 24.3, 18)	181,960 (122,434, 242,468)	13.5 (9.2, 17.5)	3488.6 (2344.6, 4641.2)	− 0.3 (− 18.8, 25.2)
Libya	6200 (4076, 8470)	19.6 (13.7, 25.2)	127 (83.1, 174.6)	19.4 (− 6.7, 57.6)	225,388 (156,923, 295,467)	13.2 (9.5, 16.7)	3921 (2681.3, 5191.8)	25.2 (0.9, 59.3)
Morocco	41,920 (25,994, 59,837)	18.4 (12, 24.7)	145.3 (89.6, 207.8)	35.7 (5, 85.8)	1,296,664 (840,201, 1,804,919)	12.8 (8.6, 17.2)	3930.3 (2543, 5491.5)	32 (2.7, 77.5)
Oman	2417 (1677, 3162)	19.5 (13.8, 24.7)	177.5 (119, 237.4)	55.7 (12.3, 150.1)	93,026 (67,425, 119,734)	10.9 (7.9, 13.8)	4401.7 (3087.8, 5712.6)	38.5 (2, 109.5)
Palestine	2816 (1806, 3960)	16.9 (11.2, 23)	131.6 (80.9, 190.9)	7.7 (− 17.3, 52.8)	95,305 (65,963, 127,230)	9.6 (6.5, 12.8)	3647.4 (2444.6, 4973.1)	6.7 (− 16.4, 43.7)
Qatar	963 (649, 1307)	21.8 (16.4, 26.3)	209.6 (137.1, 285.7)	− 8.6 (− 27.6, 19.2)	57,395 (42,382, 74,978)	12.8 (10.1, 15.2)	4904.9 (3498.2, 6432.2)	− 11.1 (− 28.2, 11.9)
Saudi Arabia	28,039 (18,987, 37,001)	21.8 (16.1, 26.7)	160.7 (109.2, 212.1)	32.2 (− 1.1, 86.4)	1,183,820 (845,229, 1,535,555)	14.2 (10.7, 17.3)	4771.5 (3389.2, 6141.8)	36.9 (4.1, 86.8)
Sudan	24,442 (15,058, 35,927)	12.1 (7.8, 16.9)	136 (82.7, 197.6)	44.6 (8.4, 123.9)	839,037 (541,166, 1,189,315)	6.6 (4.3, 9.2)	3862.4 (2483.1, 5500)	39.9 (4.8, 116.8)
Syrian Arab Republic	16,054 (9631, 24,012)	19 (12.6, 25.7)	143.8 (85.6, 216.7)	1.4 (− 24.8, 43.9)	525,266 (332,188, 746,285)	13.4 (8.8, 18.1)	3994.5 (2515.4, 5694.3)	− 2.6 (− 26.4, 35)
Tunisia	12,000 (7184, 17,969)	17.7 (11.5, 24.2)	101.7 (60.3, 153.5)	13.5 (− 16.1, 59.1)	374,208 (244,986, 532,923)	13 (8.8, 17.3)	2916.8 (1907.4, 4177.2)	19 (− 7.6, 58.1)
Turkey	80,118 (50,253, 114,779)	17.6 (11.6, 23.4)	95.2 (59.5, 136.7)	− 25.4 (− 42.5, − 4.8)	2,381,067 (1,609,701, 3,206,363)	12.1 (8.4, 15.7)	2662.6 (1804, 3599.4)	− 23.3 (− 37.6, − 5.4)
United Arab Emirates	7623 (5210, 10,335)	26.2 (20.3, 31.4)	203 (138.4, 274.6)	− 9.6 (− 28.5, 18)	373,220 (272,002, 487,534)	17.4 (13.6, 21)	5732.9 (4111.7, 7383.3)	− 2.5 (− 21.7, 26.1)
Yemen	11,780 (6337, 19,075)	6.7 (3.8, 9.9)	88.6 (45.9, 143.5)	37.8 (− 2, 131.9)	410,881 (228,751, 650,581)	3.5 (2, 5.2)	2595.6 (1422.7, 4079.9)	41.1 (− 1.6, 137.9)

DALY, Disability adjusted life year; GBD, Global Burden of Disease; ASRs, Age-standardized rates; PAF, Population Attributable Fraction; UI, Uncertainty interval.

Generated from data available from http://ghdx.healthdata.org/gbd-results-tool.

### Country level

**Figure 1:**
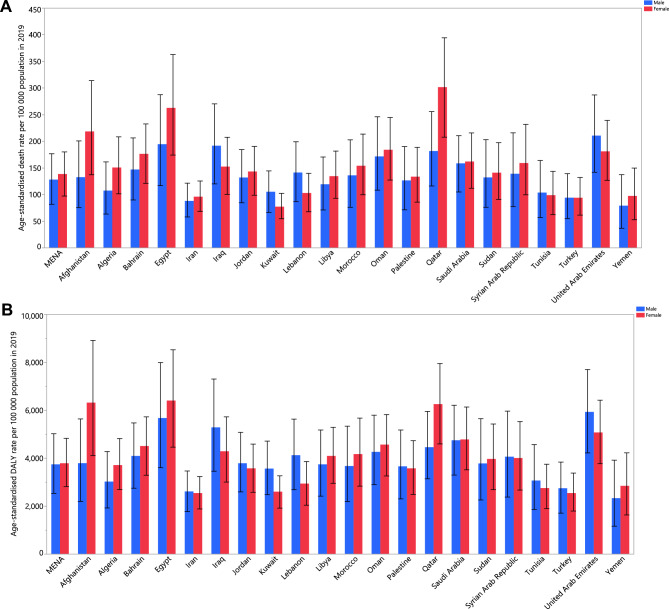
Age- standardized death (**A**) and DALY (**B**) rates (per 100,000 population) of diseases attributable to excess body weight in the Middle East and North Africa region, by sex and country. DALY = disability adjusted life years. (Generated from data available from http://ghdx.healthdata.org/gbd-results-tool).

In 2019, the proportion of all deaths linked with EBW varied markedly between countries (from 6.7% to 26.4%). Bahrain (26.4 [19.3 to 32.4]), the United Arab Emirates (26.2% [20.3 to 31.4]) and Jordan (23.4% [16.9 to 29.0]) had the three highest PAFs. Conversely, the lowest PAFs were observed in Yemen (6.7% [3.8 to 9.9]), Afghanistan (8.8% [5.7 to 12.0]) and Sudan (12.1% [7.8 to 16.9]) (Table 1). The age-standardized mortality rate linked to EBW in 2019 ranged from 88.6 to 217.7 per 100,000. In 2019, Egypt (217.7 [140.0 to 307.8]), Qatar (209.6 [137.1 to 285.7]) and the United Arab Emirates (203.0 [138.4 to 274.6]) had the three highest rates per 100,000 with  the lowest found in Yemen (88.6 [45.9 to 143.5]), Iran (91.7 [63.9 to 122.1]) and Kuwait (93.6 [62.4 to 125.4]) (Table 1). Figure 1A presents the age-standardized death rate attributable to EBW in 2019 by sex. The largest increases in the age-standardized death rate linked to EBW, between 1990 and 2019, were observed in Oman (55.7% [12.3 to 150.1]), Sudan (44.6% [8.4 to 123.9]) and Yemen (37.8% [− 2.0 to 131.9]). Conversely, the largest decreases were observed in Turkey (− 25.4% [− 42.5 to − 4.8]), Bahrain (− 23.6% [− 37.9 to − 2.5]) and Kuwait (− 23.4% [− 34.9 to − 7.4]) (Table S2). Figure S1 shows the percentage change in the age-standardized death rate, from 1990 to 2019, by sex.

The proportion of all DALYs linked with EBW in 2019 differed substantially between countries (from 3.5% to 17.4%). The United Arab Emirates (17.4% [13.6 to 21.0]), Bahrain (17.2% [12.9 to 20.9]) and Egypt (16.0% [11.3 to 20.3]) had the three highest PAFs. Conversely, the lowest PAFs were observed in Yemen (3.5% [2.0 to 5.2]), Afghanistan (4.6% [3.0 to 6.5]) and Sudan (6.6% [4.3 to 9.2]) (Table 1). In 2019, the age-standardized DALY rate due to EBW ranged from 2580.9 to 5929.6 per 100,000. Egypt (5929.6 [3973.1 to 8107.8]), the United Arab Emirates (5732.9 [4111.7 to 7383.3]) and Afghanistan (5098.6 [3230.9 to 7244.7]) had the three highest age-standardized DALY rates. Conversely, the lowest rates were found in Iran (2580.9 [1845.1 to 3337.3]), Yemen (2595.6 [1422.7 to 4079.9]) and Turkey (2662.6 [1804.0 to 3599.4]) (Table 1). Fig. 1B displays the age standardized DALY rates in 2019 by sex. The largest increases in the DALY rates, between 1990 and 2019, were observed in Yemen (41.1% [− 1.6 to 137.9]), Sudan (39.9% [4.8 to 116.8]) and Oman (38.5% [2.0 to 109.5]). Conversely, the largest decreases were observed in Turkey (− 23.3% [− 37.6 to − 5.4]), Bahrain (− 21.2% [− 35.0 to − 3.1]), and Jordan (− 19.5% [− 32.3 to − 1.0]) (Table 1). Figure S2 presents the percentage change in the age-standardized DALY rates, from 1990 to 2019, by sex.

### Age and sex patterns

**Figure 2:**
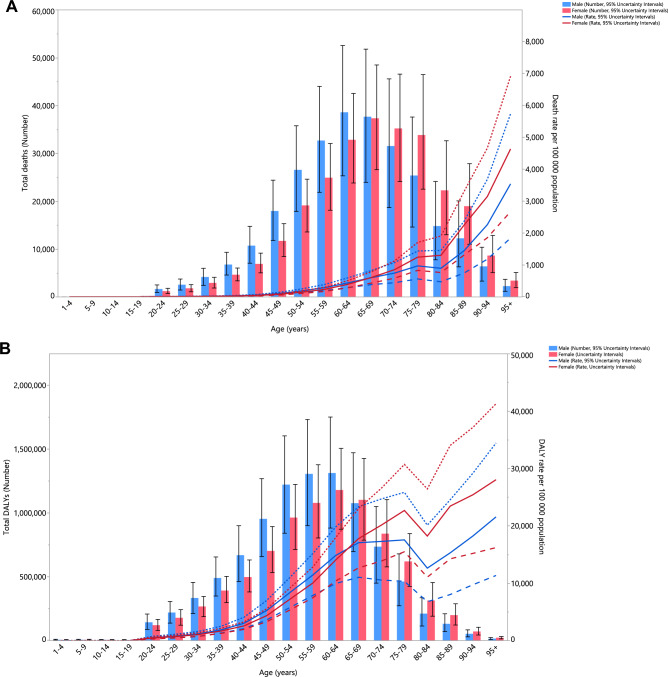
Numbers of deaths and death rate (**A**) and number of DALYs and the DALY rate (**B**) of diseases attributable to excess body weight in the Middle East and North Africa region, by age and sex in 2019. The right axis corresponds to the line chart and the left axis corresponds to the bar chart. DALY = disability adjusted life years. (Generated from data available from http://ghdx.healthdata.org/gbd-results-tool).

In 2019, the number of deaths linked to EBW in MENA peaked in the 60–64 age group for males and 65–69 age group for females. The mortality rate due to EBW increased with age and the highest values were found in the 95 + age group (Fig. 2A). Furthermore, in 2019 the number of DALYs linked with EBW in the region were highest in the 60–64 age group for both sexes. The DALY rate increased with rose age but decreased in the 80–84 age group. There were no significant  sex in the DALY rates (Fig. 2B).

### Attributable diseases

**Figure 3:**
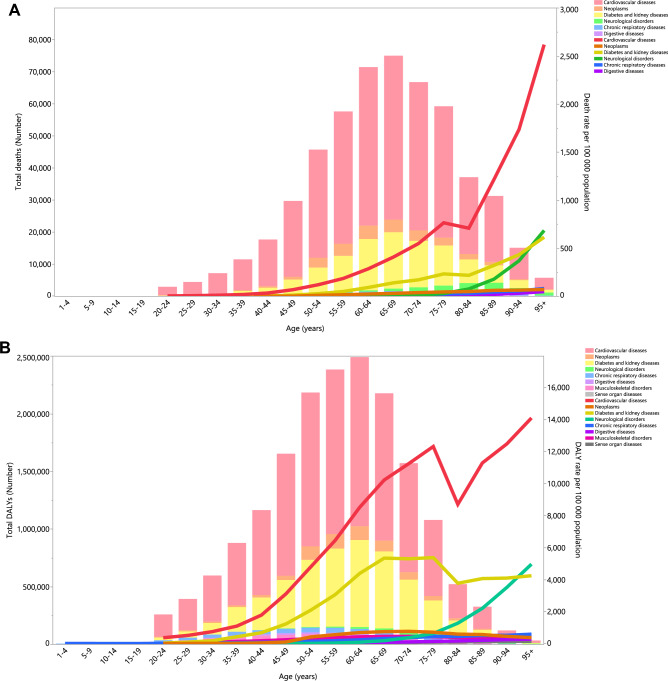
Numbers of deaths and death rate (**A**) and number of DALYs and the DALY rate (**B**) of diseases attributable to excess body weight in the Middle East and North Africa region, by age and cause in 2019. The right axis corresponds to the line chart and the left axis corresponds to the bar chart. DALY = disability adjusted life years. (Generated from data available from http://ghdx.healthdata.org/gbd-results-tool).

In 2019, cardiovascular diseases were the largest cause of deaths, with the highest death rates due to EBW observed in the 65–69 and 95^+^ categories (Fig. 3A). The death rates attributable to diabetes and kidney diseases were second in all age groups except for the 95^+^ age group (Fig. 3A). The DALY rate associated with cardiovascular diseases exhibited an increased up to the 75–79 age group, followed by a decreased in the 80–84 age group, before increasing over the subsequent age groups. In addition, the DALY rate attributable to diabetes and kidney diseases increased up to those aged 60–64 years old, reached a plateau in the 65–79 age groups, then declined in the 80–84 age group, before increasing over the remaining age groups (Fig. 3B).

### Burden of diseases attributable to EBW by the Socio-demographic Index (SDI)

**Figure 4:**
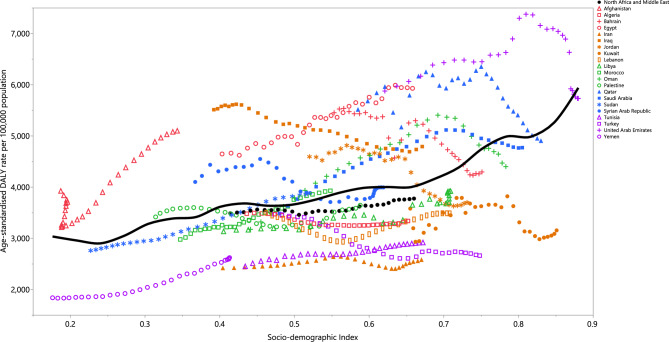
Age- standardized DALY rates of diseases attributable to excess body weight for 21 countries from 1990 to 2019, by SDI; Expected values based on the Socio-demographic Index and disease rates in all locations are shown as the black line. Each point shows the observed age- standardized DALY rate for each country. DALY = disability adjusted life years. SDI = Socio-demographic Index (Generated from data available from http://ghdx.healthdata.org/gbd-results-tool).

There was a generally positive relasionship between a country’s SDI and their corresponding age-standardized DALY rates associated with EBW. Countries like Afghanistan, Iraq, and Egypt had higher-than-expected burdens, while Yemen, Iran, Tunisia, Lebanon, Algeria, and Libya had lower-than-expected burdens. In addition, the age-standardized DALY rates increased from 1990 to 2019 in most of the MENA countries (Fig. 4).

## Discussion

This study found that about 17% of deaths and 11% of all DALYs in MENA were attributable to EBW. Egypt, the United Arab Emirates, and Afghanistan had the highest attributable burden in 2019, whereas Iran, Yemen and Turkey had the lowest. Moreover, the EBW-attributable burden increased with advancing age and had a positive association with socioeconomic development. Cardiovascular diseases accounted for the largest number of deaths and DALYs in 2019, followed by diabetes and kidney diseases.

A previous study using GBD 2017 data showed decreases in the age-standardized death (4.7%) and DALY (2.2%) rates attributable to EBW in MENA over the period from 1990 to 2017^1^. In contrast, in the Eastern Mediterranean Region, the death and DALY rates attributable to obesity increased by 11.0% and 13.9%, respectively, between 1990 and 2015^13^. Our findings of a 5.1% increase in the age-standardized death rate and an 8.3% increase in the DALY rate were slightly lower than the increases observed in the Eastern Mediterranean Region. This minor differences between these two studies may be due to the different time periods measured, differences in the geographical location (e.g., Eastern Mediterranean Region vs. MENA), the reporting of age-standardized vs. all age rates, or the use of different data sources and definitions for EBW. The MENA region includes 21 countries, as mentioned in the Methods section, and the classification is based on the GBD study. In contrast, the Eastern Mediterranean Region includes 22 countries (i.e. Afghanistan, Bahrain, Djibouti, Egypt, Iran, Iraq, Jordan, Kuwait, Lebanon, Libya, Morocco, Oman, Pakistan, Palestine, Qatar, Saudi Arabia, Somalia, Sudan, Syrian Arab Republic, Tunisia, United Arab Emirates, and Yemen) and is the classification system used by the World Health Organization. Nevertheless, there is a clear increasing trend in the age-standardized prevalence of EBW in the Eastern Mediterranean Region and globally, which suggests the potential for further increases in the EBW-attributable burden^23^. Furthermore, the age-standardized mortality and DALY rates of diseases due to EBW were higher in 2019 than they were in 2017, as reported by Dai and colleagues (133.6 vs. 109.5 per 100,000 for age-standardized death rates and 3777.2 vs. 3256.0 for age-standardized DALY rates)^1^. The results of our research underscore the importance of implementing preventive measures against EBW at various stages to mitigate the prospective disease burden lined to it.

Egypt registered the largest age-standardized mortality rate (217.7) and DALY rate (5929.6) per 100,000 in 2019, followed by Qatar, the United Arab Emirates and Afghanistan. Likewise, the GBD 2017 data indicated that Egypt had the highest age-standardized mortality (187.3 per 100,000) and DALY rates (5322.5 per 100,000) associated with high BMI^1^. Moreover, in Egypt, the prevalence of EBW among adolescents (aged 12–15 years) increased by 1.44 times between 2006 and 2011^24^. The consistent relationships between high BMI and poor health outcomes in Egypt are concerning. Previous studies have indicated that, despite Egypt’s status as a high-income country, the prevalence of obesity is high not only among the educated and wealthy, but also among the less educated and less wealthy^25^. Furthermore, many children in Egypt tend to be overnourished. Health survey data from 2000 showed that 47% of children received more than 100% of their Recommended Daily Allowance (RDA), compared with only 14% in 1995^26^. The increasingly sedentary lifestyles, coupled with the modernization and globalization of the food supply, have contributed to the rising obesity problem. Unfortunately, efforts to prevent and manage obesity are not currently on the Egyptian government’s agenda. This was revealed in qualitative data obtained from 25 interviews involving 22 organizations relevant to the obesity epidemic in Egypt^27^. Consequently, there is a need for more research and attention to generate awareness and solutions to the obesity problem in Egypt. Qatar, facing a situation akin to that of to Egypt, has recognised the significance of obesity prevention by prioritising it in the Qatari National Health Strategy (2018–22)^28^. Furthermore, in 2019, Yemen and Iran had among the lowest EBW-attributable burdens in MENA. Encouragingly, Iran has implemented the recommendations from the World Health Organization on Ending Childhood Obesity (WHO-ECHO), known as IRAN-ECHO, to prevent and control EBW in the Iranian population^29,30^. In addition, the results showed that eight countries in the Eastern Mediterranean Region, namely Afghanistan, Kuwait, Oman, the occupied Palestinian Territory, Qatar, Sudan, Syria, and Yemen, have implemented nutritional surveillance systems as part of the WHO Recommended Policies and Interventions on Healthy Diets. This initiative aims to improve nutrition in order to reduce the prevalence of EBW^31^.

Previous studies have consistently shown that the burden of diseases attributed to EBW was slightly higher in women compared to men, both at the global and regional levels, although these differences were relatively modest^1^. Moreover, research has highlighted the fact that in Middle Eastern countries and the broader Eastern Mediterranean Region, females exhibited a greater prevalence of EBW^13,32^. Building upon these established findings, the present study also found that the age-standardized mortality and DALY rates were higher among females, with these disparities being particularly notable among older adults. Furthermore, it is noteworthy that the 60-64 age group had the largest number of DALYs which underscores the importance of addressing this issue among those nearing retirement. In addition, the highest age-standardized rates due to EBW were identified in individuals aged 95 years and older, further reinforcing the importance of preventive measures and interventions targeting this group. These findings were also in accordance with research by Dai et al. using GBD 2017 data^1^. In summary, it is advisable to implement preventive measures during adolescence and among young adults to reduce the burden attributable to EBW later in life.

The current study showed that cardiovascular diseases had the highest death and DALY numbers and rates, which is consistent with the findings reported in 2017^1^. Diabetes and kidney disease accounted for the second highest number of deaths and death rates. It’s important to note that EBW serves as a well-established risk factors for all of these conditions, with particularly strong associations with cardiovascular diseases and diabetes. Unfortunately, lipid accumulation and fatty streaks develop in young adults^33^, with obesity accelerating atherosclerotic changes through mechanisms such as insulin resistance and inflammation^34^. The presence of obesity can lead to the exacerbation of metabolic cardiovascular risk factors, such as elevated blood pressure, dyslipidemia and hyperglycemia, all of which play a significant role in the development and progression of various diseases in affected individuals. For obesity-related kidney diseases, mechanisms include activation of the renin–angiotensin–aldosterone system, systemic inflammation, endothelial dysfunction, adipokine release, insulin resistance, and hypertension^35^. Importantly, these modifiable risk factors can be effectively prevented and managed through improved diet and lifestyle, as shown in clinical trials^36–38^, underscoring the potential of these strategies to mitigate the disease burden associated with excess body weight.

Several preventive strategies for obesity have been developed, primarily in high-income countries like Australia^39^, the USA^40^, and the UK^41^, which focus on improved nutrition, physical activity, optimal sleep and stress reduction. In the MENA region, prevention strategies have been developed based on recommendations provided by the World Obesity Federation^42^. In addition to understanding the problem of obesity and its underlying causes, MENA prevention strategies are generally in line with those of high-income countries. However, countries in the MENA region face several different obstacles compared to those in high-income countries. These include a severely limited number of health professionals, inconsistencies in training and approaches to obesity prevention, a lack of formal sponsored guidelines, poor public transport systems, and insufficient funding for obesity research. The frameworks developed by the WHO guide obesity prevention strategies in the MENA region, encompassing both individual and societal level changes. These changes include limiting energy intake from total fats, increasing consumption of fruit, vegetables, legumes, whole grains, and nuts, restricting sugar intake, and promoting regular physical activity^42^. Public health prevention at the national and regional level is also being considered, including the use of mandatory food labeling, marketing restrictions, taxation of certain foods and beverages, and responsible marketing practices, especially when targeting children. The prevention of excess body weight requires multi-factorial strategies, but individual behavior change is also required.

## Strengths and limitations

This study has several strengths, including the use of advanced analyses to study the burden of diseases linked to EBW, as well as its focus on the MENA region over a 30-year period. However, it also has several limitations. One of these limitations pertains to the absence of high-quality data in several low- and middle-income countries in the MENA region, a circumstance that may introduce the potential for either underestimating or over-estimating the burden associated with EBW. Moreover, the GBD study employed a different modeling system to estimate the burden in countries with no data available, meaning that the findings are based on estimations rather than real data. Secondly, certain diseases attributable to EBW, such as reproductive disorders (e.g. infertility)^43^, dental disorders (e.g. periodontitis)^44^ and psychiatric disorders (e.g. depression)^45^, were not reported in the present study. Thirdly, the study’s definition of overweight and obesity solely relied on BMI, a practical but imprecise measure, whereas more sophisticated methods like computed tomography, magnetic resonance imaging or dual-energy x-ray absorptiometry, which can offer greater accuracy, were not incorporated in the present study. Finally, race/ethnicity specific data were not reported, despite the known relationship between EBW and socioeconomic factors like race/ethnicity^46^. Additionally, data for sub-geography were not available for all 21 countries in MENA, making it impossible to report the attributable burden by sub-geography. Furthermore, in MENA there are large differences in the level of economic development, which is a predisposing factor for EBW. These shortcomings should be addressed to ensure more comprehensive and accurate estimations in the future.

## Conclusions

The burden of disease associated with EBW has increased in MENA, with substantial differences between the different countries in the region. Efforts to develop and implement effective preventive programs are imperative on both the regional and national scales, especially in those countries categorized as having higher levels of socioeconomic development. Furthermore, there is a demand for primary and secondary prevention measures aimed at addressing diseases associated with EBW, especially cardiovascular diseases, neoplasms, diabetes, and kidney diseases.

### Ethics approval and consent to participate

This study was approved by the Shahid Beheshti University of Medical Sciences, Tehran, Iran (IR.SBMU.PHNS.REC.1401.101) and the Tabriz University of Medical Sciences, Tabriz, Iran (IR.TBZMED.REC.1401.957).

### Supplementary Information

Supplementary Figure 1.

Supplementary Figure 2.

Supplementary Table 1.

Supplementary Table 2.

Supplementary Table 3.

Supplementary Table 4.

## Data Availability

The data used for these analyses are all publicly available at https://vizhub.healthdata.org/gbd-results/.
