# Supplementary material for: Burden of diseases attributable to excess body weight in the Middle East and North Africa region, 1990–2019
Source: Sci Rep. 2023 Nov 20;13:20338. doi: 10.1038/s41598-023-46702-y (PMC10663478; doi:10.1038/s41598-023-46702-y)
Supplement: Supplementary file 2 — Supplementary Figure 2. [file 41598_2023_46702_MOESM2_ESM.pdf]

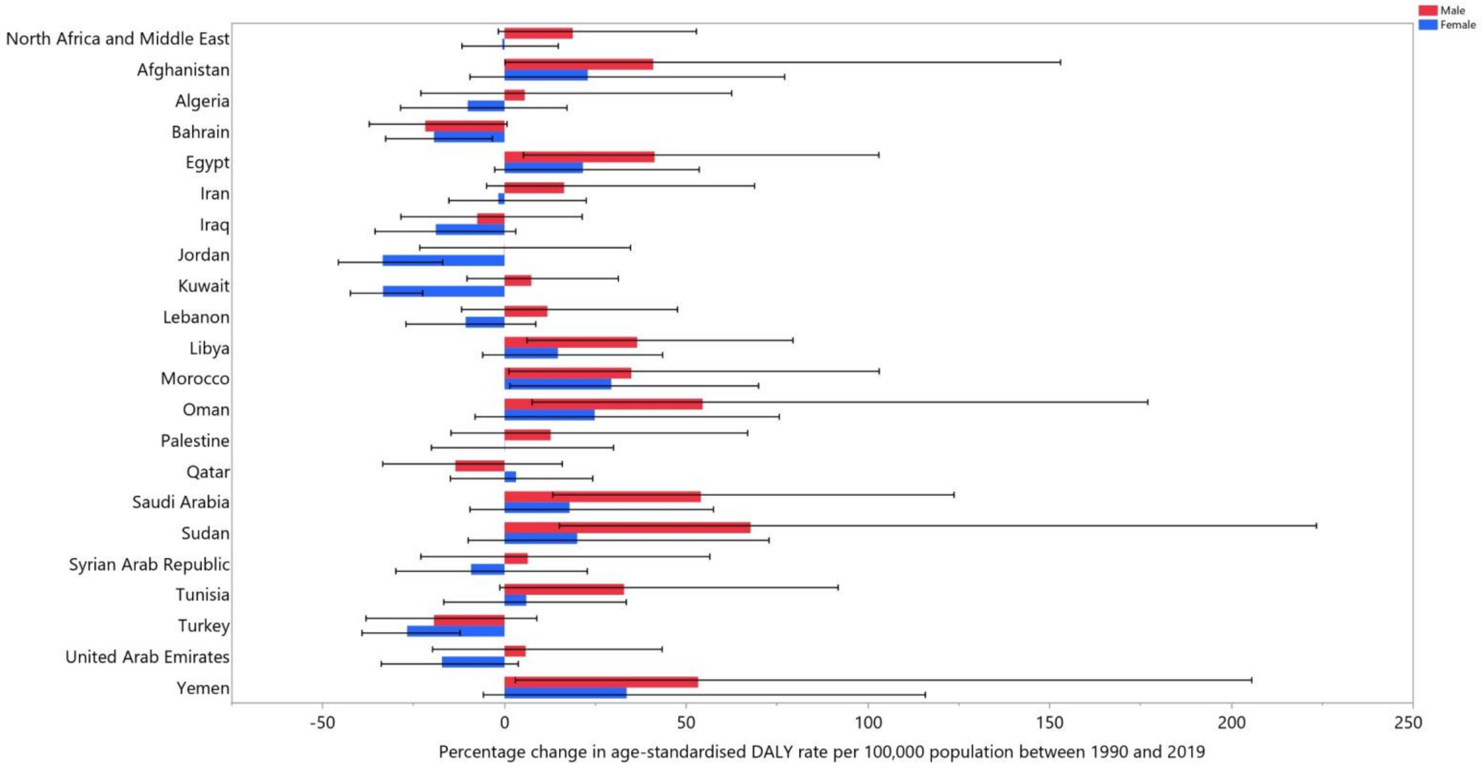

Figure S2: The percentage change in the age- standardized DALYs rate of diseases attributable to excess body weight in the Middle East and North Africa region from 1990 to 2019, by sex and country. DALY= disability adjusted life years. (Generated from data available from http://ghdx.healthdata.org/gbd-results-tool).
