# Supplementary material for: Burden of diseases attributable to excess body weight in the Middle East and North Africa region, 1990–2019
Source: Sci Rep. 2023 Nov 20;13:20338. doi: 10.1038/s41598-023-46702-y (PMC10663478; doi:10.1038/s41598-023-46702-y)
Supplement: Supplementary file 3 — Supplementary Table 1. [file 41598_2023_46702_MOESM3_ESM.doc]

| **Table S1: Deaths attributable to excess body weight in the Middle East and North Africa region in 2019 by sex**  **(Generated from data available from http://ghdx.healthdata.org/gbd-results-tool)** | | | | | | | | |
| --- | --- | --- | --- | --- | --- | --- | --- | --- |
|  | **Male** | | | | **Female** | | | |
|  | **No**  **(95% UI)** | **PAF**  **(95% UI)** | **ASRs per 100,000 (95% UI)** | **% change in ASRs per 100,000**  **1990-2019** | **No**  **(95% UI)** | **PAF**  **(95% UI)** | **ASRs per 100,000 (95% UI)** | **% change in ASRs per 100,000**  **1990-2019** |
| **North Africa and Middle East** | **272291 (177828 , 368486)** | **15.5 (10.3 , 20.8)** | **128.1 (81.8 , 176.7)** | **15.1 (-4.9 , 50.4)** | **266157 (190448 , 343961)** | **19.8 (14.4 , 25.1)** | **138.6 (97.2 , 180.1)** | **-2.2 (-14.5 , 14.1)** |
| **Afghanistan** | **7986 (4506 , 12188)** | **6.3 (3.7 , 9.2)** | **132.7 (75.5 , 200.8)** | **40.6 (-1.1 , 154.1)** | **14062 (8978 , 20254)** | **11.2 (7.6 , 15)** | **218.5 (137.4 , 313.9)** | **26.3 (-8 , 89.5)** |
| **Algeria** | **16530 (10046 , 24298)** | **15.5 (9.8 , 21.7)** | **107.5 (63.5 , 161.4)** | **0.2 (-28.2 , 61.3)** | **19557 (13397 , 26184)** | **20.7 (14.7 , 26.8)** | **150.9 (101.5 , 208.4)** | **-7 (-26.6 , 29.1)** |
| **Bahrain** | **669 (440 , 913)** | **25.5 (18.3 , 31.9)** | **147.1 (89.9 , 206.4)** | **-25.5 (-42.3 , -0.3)** | **458 (327 , 596)** | **27.8 (20.7 , 33.6)** | **176.5 (121.1 , 232.6)** | **-20.3 (-34.9 , -0.1)** |
| **Egypt** | **67152 (41079 , 97162)** | **20.6 (13.3 , 27.6)** | **194.6 (117 , 287.6)** | **36.3 (-1.3 , 98.4)** | **63190 (42935 , 86832)** | **26.8 (19.3 , 33.3)** | **262.8 (174.2 , 362.9)** | **28.5 (1.1 , 65)** |
| **Iran** | **30812 (20622 , 42064)** | **13.7 (9.2 , 18.6)** | **88.1 (57.9 , 121.5)** | **9.1 (-10.8 , 57.2)** | **30603 (22263 , 39710)** | **18.4 (13.4 , 23.5)** | **96.1 (68.5 , 125.6)** | **-0.7 (-16.6 , 28.3)** |
| **Iraq** | **20266 (12867 , 28504)** | **19.4 (12.7 , 25.5)** | **191.7 (120.1 , 270.4)** | **-3.1 (-26 , 28.6)** | **16215 (10838 , 22179)** | **21.5 (15.2 , 27.4)** | **152.5 (100.2 , 207.6)** | **-19.6 (-37.3 , 4.3)** |
| **Jordan** | **4091 (2679 , 5639)** | **22.1 (15.4 , 28)** | **132 (84.8 , 184.5)** | **-2.2 (-26.7 , 37)** | **3458 (2419 , 4517)** | **25.1 (18.7 , 30.5)** | **143.2 (98.9 , 190.4)** | **-35 (-48.5 , -15.9)** |
| **Kuwait** | **1647 (1090 , 2237)** | **23.3 (16.6 , 29.4)** | **105.2 (66.4 , 144.5)** | **0.5 (-19.7 , 29.5)** | **670 (481 , 871)** | **22.7 (17 , 27.7)** | **77.4 (54.6 , 102.2)** | **-45.3 (-54.2 , -33.6)** |
| **Lebanon** | **3270 (2004 , 4603)** | **17.3 (10.7 , 24)** | **141.4 (86.8 , 199.3)** | **9.6 (-14.6 , 46.3)** | **2895 (1906 , 3930)** | **19.3 (12.7 , 25.7)** | **102.9 (67.9 , 139.9)** | **-18 (-35.3 , 1.4)** |
| **Libya** | **3016 (1832 , 4297)** | **16.8 (11 , 22.6)** | **119.3 (71.1 , 170.6)** | **30.5 (-1.8 , 81)** | **3184 (2219 , 4293)** | **23.2 (17 , 29.1)** | **134.5 (92.9 , 181.7)** | **9.8 (-13.4 , 42.6)** |
| **Morocco** | **19614 (11063 , 29070)** | **15.8 (9.5 , 22.5)** | **136.2 (76.1 , 202.8)** | **39.1 (1.8 , 108.4)** | **22306 (14672 , 30913)** | **21.4 (14.7 , 28.3)** | **154 (99.8 , 213.6)** | **32.8 (2.8 , 78.3)** |
| **Oman** | **1357 (911 , 1869)** | **17.2 (11.8 , 22.3)** | **171.6 (108.3 , 246.2)** | **77 (21.4 , 230.6)** | **1060 (754 , 1374)** | **23.5 (17.1 , 29.3)** | **184.2 (127.3 , 244.8)** | **40.7 (0.6 , 105.7)** |
| **Palestine** | **1371 (842 , 1987)** | **15.4 (9.7 , 21.5)** | **126.5 (71.4 , 190.1)** | **10.4 (-16.7 , 69.3)** | **1444 (959 , 1990)** | **18.8 (12.8 , 25)** | **133.6 (85.8 , 188.7)** | **3.5 (-20.4 , 44)** |
| **Qatar** | **683 (449 , 950)** | **20.5 (15.2 , 25.2)** | **181.8 (116.1 , 255.8)** | **-13.8 (-35.9 , 22.4)** | **279 (202 , 367)** | **25.7 (19.9 , 30.5)** | **301.8 (207.7 , 394.3)** | **21.6 (-2.2 , 51.8)** |
| **Saudi Arabia** | **17228 (11472 , 23145)** | **20.1 (14.4 , 25.2)** | **158.6 (105 , 210.6)** | **48.5 (8.2 , 122.6)** | **10811 (7642 , 14287)** | **25.2 (19.2 , 30.1)** | **162.1 (111.9 , 215.7)** | **15.5 (-14.2 , 59.6)** |
| **Sudan** | **12917 (7415 , 19738)** | **11 (6.6 , 16.1)** | **132.2 (76.2 , 203.1)** | **72.6 (18.2 , 234.2)** | **11525 (7485 , 16339)** | **13.6 (9.2 , 18.3)** | **141 (90.8 , 197.5)** | **24.7 (-5 , 80.8)** |
| **Syrian Arab Republic** | **8438 (4656 , 13029)** | **17.4 (10.8 , 24.3)** | **139.1 (77.4 , 216)** | **10.3 (-21.8 , 65.5)** | **7616 (4929 , 10914)** | **21.1 (14.6 , 27.5)** | **159.2 (99.6 , 231.8)** | **-0.4 (-24.6 , 37.3)** |
| **Tunisia** | **5968 (3274 , 9417)** | **15.6 (9.4 , 22)** | **103.7 (56.9 , 164)** | **26.6 (-10.9 , 93.5)** | **6032 (3814 , 8748)** | **20.5 (13.8 , 27.2)** | **98.9 (62.3 , 143.7)** | **1.6 (-24.4 , 36.4)** |
| **Turkey** | **37388 (22079 , 54899)** | **15.1 (9.5 , 20.5)** | **94.2 (54.9 , 139.3)** | **-21 (-42.1 , 10.6)** | **42730 (27951 , 59892)** | **20.5 (14.1 , 26.7)** | **94.1 (61.5 , 132.1)** | **-28.9 (-44.9 , -11.1)** |
| **United Arab Emirates** | **6308 (4263 , 8619)** | **26.2 (19.8 , 31.9)** | **210.7 (141.9 , 287)** | **1.3 (-24.1 , 39.5)** | **1315 (960 , 1733)** | **26 (20.8 , 30.3)** | **181.1 (126.9 , 239.4)** | **-25.2 (-41.3 , -1.8)** |
| **Yemen** | **5303 (2500 , 9106)** | **5.4 (2.8 , 8.5)** | **79.2 (36.7 , 137.5)** | **52.3 (1.3 , 224.4)** | **6477 (3610 , 9970)** | **8.5 (4.9 , 12.1)** | **97.5 (52.8 , 149.7)** | **31.2 (-6.4 , 112.5)** |

ASRs: Age-standardized rates; PAF: Population Attributable Fraction; UI: Uncertainty interval
