# Supplementary material for: Burden of diseases attributable to excess body weight in the Middle East and North Africa region, 1990–2019
Source: Sci Rep. 2023 Nov 20;13:20338. doi: 10.1038/s41598-023-46702-y (PMC10663478; doi:10.1038/s41598-023-46702-y)
Supplement: Supplementary file 4 — Supplementary Table 2. [file 41598_2023_46702_MOESM4_ESM.doc]

| **Table S2: Deaths attributable to excess body weight in the Middle East and North Africa region in 1990 and 2019**  **(Generated from data available from http://ghdx.healthdata.org/gbd-results-tool)** | | | | | | | |
| --- | --- | --- | --- | --- | --- | --- | --- |
|  | **1990** | | | **2019** | | | **% change in ASRs per 100,000**  **1990-2019** |
|  | **No**  **(95% UI)** | **PAF**  **(95% UI)** | **ASRs per 100,000 (95% UI)** | **No**  **(95% UI)** | **PAF**  **(95% UI)** | **ASRs per 100,000 (95% UI)** |
| **North Africa and Middle East** | **207355 (132867 , 289775)** | **8.2 (5.2 , 11.5)** | **127.1 (79.2 , 181.3)** | **538448 (369917 , 712329)** | **17.4 (12.2 , 22.5)** | **133.6 (90 , 179)** | **5.1 (-9 , 25.9)** |
| **Afghanistan** | **9518 (4739 , 15419)** | **5.2 (2.7 , 8.2)** | **132.7 (63.9 , 217.1)** | **22048 (13613 , 32635)** | **8.8 (5.7 , 12)** | **177.3 (109.1 , 256.1)** | **33.6 (-1.9 , 103.3)** |
| **Algeria** | **14353 (8613 , 21618)** | **9.3 (5.8 , 13.6)** | **131.8 (75.3 , 203.2)** | **36087 (23393 , 50726)** | **17.9 (12.2 , 24.1)** | **125.2 (80.3 , 177.3)** | **-5 (-27 , 32.4)** |
| **Bahrain** | **339 (223 , 452)** | **17.2 (11.7 , 22.5)** | **211.6 (136.1 , 293)** | **1127 (768 , 1504)** | **26.4 (19.3 , 32.4)** | **161.7 (107.1 , 219.5)** | **-23.6 (-37.9 , -2.5)** |
| **Egypt** | **48450 (30605 , 67444)** | **10.4 (6.6 , 14.7)** | **173.8 (107.3 , 248.6)** | **130342 (83824 , 184741)** | **23.2 (16 , 30)** | **217.7 (140 , 307.8)** | **25.3 (-3.4 , 64.9)** |
| **Iran** | **21127 (12854 , 30040)** | **5.9 (3.6 , 8.5)** | **89.2 (52.9 , 131.7)** | **61415 (43342 , 81358)** | **15.7 (11.1 , 20.7)** | **91.7 (63.9 , 122.1)** | **2.8 (-13.1 , 32.6)** |
| **Iraq** | **14813 (9288 , 20590)** | **12.5 (7.7 , 17)** | **194.5 (119.3 , 272.1)** | **36481 (23496 , 51146)** | **20.3 (13.7 , 26.3)** | **172 (111.2 , 237.2)** | **-11.5 (-30.5 , 11)** |
| **Jordan** | **2117 (1401 , 2826)** | **14.1 (9.5 , 18.6)** | **178.4 (114.9 , 244.4)** | **7548 (5303 , 9857)** | **23.4 (16.9 , 29)** | **137.1 (94.3 , 183.4)** | **-23.1 (-36.3 , -2.4)** |
| **Kuwait** | **732 (515 , 937)** | **13.7 (9.6 , 17.5)** | **122.2 (81.3 , 161.8)** | **2317 (1596 , 3014)** | **23.1 (16.8 , 29)** | **93.6 (62.4 , 125.4)** | **-23.4 (-34.9 , -7.4)** |
| **Lebanon** | **2722 (1650 , 3944)** | **12.1 (7.5 , 17.1)** | **128.1 (75.9 , 185.7)** | **6165 (3951 , 8493)** | **18.2 (11.7 , 24.6)** | **120.6 (77.6 , 166.4)** | **-5.9 (-24.3 , 18)** |
| **Libya** | **1945 (1265 , 2700)** | **9.9 (6.5 , 13.4)** | **106.4 (67.5 , 150.1)** | **6200 (4076 , 8470)** | **19.6 (13.7 , 25.2)** | **127 (83.1 , 174.6)** | **19.4 (-6.7 , 57.6)** |
| **Morocco** | **14146 (8488 , 20962)** | **8 (4.8 , 11.6)** | **107.1 (62.4 , 161.4)** | **41920 (25994 , 59837)** | **18.4 (12 , 24.7)** | **145.3 (89.6 , 207.8)** | **35.7 (5 , 85.8)** |
| **Oman** | **757 (415 , 1176)** | **7.7 (4.5 , 11.2)** | **114.1 (60 , 182.3)** | **2417 (1677 , 3162)** | **19.5 (13.8 , 24.7)** | **177.5 (119 , 237.4)** | **55.7 (12.3 , 150.1)** |
| **Palestine** | **1039 (561 , 1656)** | **8.5 (4.8 , 12.8)** | **122.2 (63.5 , 197.3)** | **2816 (1806 , 3960)** | **16.9 (11.2 , 23)** | **131.6 (80.9 , 190.9)** | **7.7 (-17.3 , 52.8)** |
| **Qatar** | **201 (134 , 270)** | **15.2 (10.4 , 19.6)** | **229.4 (147 , 313.1)** | **963 (649 , 1307)** | **21.8 (16.4 , 26.3)** | **209.6 (137.1 , 285.7)** | **-8.6 (-27.6 , 19.2)** |
| **Saudi Arabia** | **7049 (4315 , 10349)** | **8.5 (5.5 , 11.9)** | **121.6 (73.5 , 178.7)** | **28039 (18987 , 37001)** | **21.8 (16.1 , 26.7)** | **160.7 (109.2 , 212.1)** | **32.2 (-1.1 , 86.4)** |
| **Sudan** | **8856 (4445 , 14191)** | **3.5 (1.8 , 5.4)** | **94.1 (47.2 , 153)** | **24442 (15058 , 35927)** | **12.1 (7.8 , 16.9)** | **136 (82.7 , 197.6)** | **44.6 (8.4 , 123.9)** |
| **Syrian Arab Republic** | **7516 (4338 , 11295)** | **10.2 (6.2 , 14.8)** | **141.9 (80.9 , 214.4)** | **16054 (9631 , 24012)** | **19 (12.6 , 25.7)** | **143.8 (85.6 , 216.7)** | **1.4 (-24.8 , 43.9)** |
| **Tunisia** | **4148 (2546 , 6095)** | **9.6 (6 , 13.9)** | **89.6 (52.9 , 133.8)** | **12000 (7184 , 17969)** | **17.7 (11.5 , 24.2)** | **101.7 (60.3 , 153.5)** | **13.5 (-16.1 , 59.1)** |
| **Turkey** | **43299 (27307 , 60239)** | **11.1 (7.1 , 15.4)** | **127.6 (79.5 , 179.5)** | **80118 (50253 , 114779)** | **17.6 (11.6 , 23.4)** | **95.2 (59.5 , 136.7)** | **-25.4 (-42.5 , -4.8)** |
| **United Arab Emirates** | **911 (618 , 1245)** | **15.8 (11 , 20.6)** | **224.6 (152 , 304.6)** | **7623 (5210 , 10335)** | **26.2 (20.3 , 31.4)** | **203 (138.4 , 274.6)** | **-9.6 (-28.5 , 18)** |
| **Yemen** | **3179 (1248 , 5816)** | **2.2 (0.9 , 3.9)** | **64.3 (24.5 , 120.1)** | **11780 (6337 , 19075)** | **6.7 (3.8 , 9.9)** | **88.6 (45.9 , 143.5)** | **37.8 (-2 , 131.9)** |

ASRs: Age-standardized rates; PAF: Population Attributable Fraction; UI: Uncertainty interval
