# Supplementary material for: Burden of diseases attributable to excess body weight in the Middle East and North Africa region, 1990–2019
Source: Sci Rep. 2023 Nov 20;13:20338. doi: 10.1038/s41598-023-46702-y (PMC10663478; doi:10.1038/s41598-023-46702-y)
Supplement: Supplementary file 5 — Supplementary Table 3. [file 41598_2023_46702_MOESM5_ESM.doc]

| **Table S3: DALYs attributable to excess body weight in the Middle East and North Africa region in 2019 by sex**  **(Generated from data available from http://ghdx.healthdata.org/gbd-results-tool)** | | | | | | | | |
| --- | --- | --- | --- | --- | --- | --- | --- | --- |
|  | **Male** | | | | **Female** | | | |
|  | **No**  **(95% UI)** | **PAF**  **(95% UI)** | **ASRs per 100,000 (95% UI)** | **% change in ASRs per 100,000**  **1990-2019** | **No**  **(95% UI)** | **PAF**  **(95% UI)** | **ASRs per 100,000 (95% UI)** | **% change in ASRs per 100,000**  **1990-2019** |
| **North Africa and Middle East** | **9339974 (6451196 , 12395477)** | **10.6 (7.3 , 13.8)** | **3747.8 (2535.7 , 5023.4)** | **18.8 (-1.7 , 52.8)** | **8547760 (6395161 , 10832307)** | **11.3 (8.4 , 14.1)** | **3793.2 (2815.1 , 4834)** | **-0.6 (-11.7 , 14.8)** |
| **Afghanistan** | **287498 (163030 , 440483)** | **3.3 (1.9 , 4.9)** | **3794.6 (2199 , 5642.2)** | **40.9 (0.1 , 153)** | **503936 (330366 , 720845)** | **6 (4 , 8.3)** | **6322.8 (4112.6 , 8914.9)** | **22.9 (-9.5 , 77.1)** |
| **Algeria** | **551114 (355336 , 776180)** | **10.8 (7.2 , 14.6)** | **3028.2 (1926.8 , 4279.5)** | **5.6 (-23 , 62.5)** | **625902 (459632 , 807804)** | **12.5 (9.2 , 15.7)** | **3714.8 (2697.6 , 4821)** | **-10.1 (-28.6 , 17.2)** |
| **Bahrain** | **31327 (21988 , 41306)** | **18.1 (13.4 , 22.3)** | **4095.9 (2750.4 , 5476.1)** | **-21.8 (-37.2 , 0.7)** | **17651 (13167 , 22473)** | **15.7 (12.1 , 18.9)** | **4510.2 (3291.1 , 5728.9)** | **-19.4 (-32.7 , -3.3)** |
| **Egypt** | **2234433 (1435481 , 3135139)** | **15.5 (10.3 , 20.2)** | **5679.2 (3607.3 , 7995.1)** | **41.3 (5.2 , 103)** | **1982933 (1406576 , 2617522)** | **16.6 (12.3 , 20.7)** | **6410.1 (4464.2 , 8525.8)** | **21.6 (-2.7 , 53.6)** |
| **Iran** | **1037098 (712749 , 1364782)** | **9.8 (6.9 , 12.9)** | **2617.7 (1778.2 , 3468.9)** | **16.4 (-4.9 , 68.8)** | **952359 (710774 , 1205407)** | **10.3 (7.7 , 13.1)** | **2545.9 (1886.3 , 3238.4)** | **-1.7 (-15.3 , 22.5)** |
| **Iraq** | **685148 (456871 , 945686)** | **12.3 (8.4 , 16.1)** | **5291 (3457.9 , 7306.9)** | **-7.5 (-28.5 , 21.4)** | **551649 (387894 , 736777)** | **11.7 (8.4 , 15.1)** | **4293 (3007 , 5727.8)** | **-18.9 (-35.6 , 3.1)** |
| **Jordan** | **149592 (104102 , 198826)** | **13.2 (9.4 , 16.6)** | **3792.7 (2597.8 , 5081.8)** | **-0.1 (-23.3 , 34.7)** | **113026 (83534 , 143173)** | **11.3 (8.6 , 13.7)** | **3581.2 (2581.2 , 4593)** | **-33.5 (-45.7 , -17)** |
| **Kuwait** | **73640 (52682 , 95412)** | **17.2 (12.9 , 21.1)** | **3568.7 (2478.1 , 4717.4)** | **7.4 (-10.3 , 31.3)** | **35018 (26042 , 44092)** | **10.6 (8.4 , 12.7)** | **2607.1 (1919.1 , 3268.1)** | **-33.4 (-42.4 , -22.5)** |
| **Lebanon** | **98356 (64170 , 134385)** | **14.2 (9.2 , 18.7)** | **4129.4 (2689.1 , 5635.3)** | **11.8 (-11.8 , 47.6)** | **83604 (58005 , 109642)** | **12.7 (8.9 , 16.3)** | **2943.4 (2042.1 , 3863.4)** | **-10.7 (-27.1 , 8.6)** |
| **Libya** | **112327 (74311 , 154711)** | **12.4 (8.4 , 16)** | **3744.8 (2421.4 , 5180.8)** | **36.5 (6.2 , 79.4)** | **113061 (82281 , 145752)** | **14.2 (10.7 , 17.5)** | **4098.6 (2954.8 , 5292)** | **14.7 (-6 , 43.5)** |
| **Morocco** | **605909 (365314 , 874499)** | **11.8 (7.3 , 16.4)** | **3677.7 (2196.2 , 5340.1)** | **34.9 (1.2 , 103.1)** | **690755 (469853 , 937182)** | **13.9 (9.7 , 18.2)** | **4175.2 (2837.4 , 5677.6)** | **29.4 (1.5 , 69.9)** |
| **Oman** | **56763 (39791 , 75208)** | **10.4 (7.4 , 13.4)** | **4269.1 (2900.3 , 5799.9)** | **54.5 (7.6 , 177)** | **36263 (27083 , 45389)** | **11.7 (8.7 , 14.8)** | **4568.4 (3260.6 , 5823.2)** | **24.8 (-8.1 , 75.6)** |
| **Palestine** | **49507 (32554 , 68713)** | **9.5 (6.3 , 12.9)** | **3661.5 (2306.6 , 5181)** | **12.7 (-14.7 , 66.9)** | **45798 (32356 , 59752)** | **9.7 (6.7 , 12.7)** | **3578.8 (2494.2 , 4735.5)** | **0.1 (-20.1 , 30)** |
| **Qatar** | **43128 (31481 , 56977)** | **13.1 (10.3 , 15.7)** | **4463.3 (3143.5 , 5954.4)** | **-13.5 (-33.5 , 15.9)** | **14267 (10833 , 18051)** | **11.8 (9.5 , 13.9)** | **6260.2 (4598.9 , 7954)** | **3.2 (-14.9 , 24.3)** |
| **Saudi Arabia** | **723299 (507093 , 953779)** | **14.1 (10.4 , 17.4)** | **4751.3 (3298.8 , 6212.5)** | **54 (13.3 , 123.7)** | **460521 (345310 , 588127)** | **14.5 (11.3 , 17.4)** | **4786.9 (3517.9 , 6139.5)** | **17.9 (-9.5 , 57.5)** |
| **Sudan** | **437285 (259268 , 648005)** | **6.4 (3.8 , 9.3)** | **3784.9 (2259.1 , 5653.3)** | **67.7 (15.1 , 223.4)** | **401752 (272647 , 556198)** | **6.9 (4.7 , 9.5)** | **3972.6 (2691.4 , 5430.3)** | **20 (-10 , 72.8)** |
| **Syrian Arab Republic** | **276116 (162020 , 406098)** | **13.3 (8.4 , 18.5)** | **4066.3 (2382.6 , 5968.4)** | **6.4 (-23 , 56.5)** | **249150 (166114 , 342671)** | **13.4 (9.5 , 17.6)** | **4011.7 (2674.1 , 5532.2)** | **-9.2 (-29.9 , 22.8)** |
| **Tunisia** | **194509 (118836 , 285425)** | **12.8 (8.2 , 17.5)** | **3073.3 (1863 , 4571.5)** | **32.9 (-1.3 , 91.8)** | **179699 (122717 , 245396)** | **13.2 (9.3 , 17.2)** | **2756.4 (1899.6 , 3752.9)** | **6 (-16.7 , 33.5)** |
| **Turkey** | **1192578 (754316 , 1661324)** | **11.8 (7.7 , 15.5)** | **2747.3 (1712.5 , 3840.3)** | **-19.4 (-38.1 , 8.9)** | **1188489 (841425 , 1575605)** | **12.5 (9 , 16)** | **2548.7 (1797.2 , 3383.8)** | **-26.8 (-39.2 , -12.2)** |
| **United Arab Emirates** | **305186 (218987 , 403512)** | **18.5 (14.2 , 22.5)** | **5935.3 (4225.9 , 7703.1)** | **5.8 (-19.8 , 43.4)** | **68035 (51078 , 87568)** | **13.7 (11 , 16.4)** | **5076 (3772 , 6422.7)** | **-17.2 (-33.9 , 3.8)** |
| **Yemen** | **185671 (94198 , 310855)** | **3 (1.6 , 4.8)** | **2335.1 (1164.4 , 3923.2)** | **53.3 (3 , 205.6)** | **225210 (132192 , 335561)** | **4.1 (2.5 , 5.9)** | **2849.5 (1635.8 , 4227.4)** | **33.6 (-5.8 , 115.8)** |

ASRs: Age-standardized rates; PAF: Population Attributable Fraction; UI: Uncertainty interval
