# Supplementary material for: Burden of diseases attributable to excess body weight in the Middle East and North Africa region, 1990–2019
Source: Sci Rep. 2023 Nov 20;13:20338. doi: 10.1038/s41598-023-46702-y (PMC10663478; doi:10.1038/s41598-023-46702-y)
Supplement: Supplementary file 6 — Supplementary Table 4. [file 41598_2023_46702_MOESM6_ESM.doc]

| **Table S4: DALYs attributable to excess body weight in the Middle East and North Africa region in 1990 and 2019**  **(Generated from data available from http://ghdx.healthdata.org/gbd-results-tool)** | | | | | | | |
| --- | --- | --- | --- | --- | --- | --- | --- |
|  | **1990** | | | **2019** | | | **% change in ASRs per 100,000**  **1990-2019** |
|  | **No**  **(95% UI)** | **PAF**  **(95% UI)** | **ASRs per 100,000 (95% UI)** | **No**  **(95% UI)** | **PAF**  **(95% UI)** | **ASRs per 100,000 (95% UI)** |
| **North Africa and Middle East** | **6673555 (4372832 , 9136197)** | **4 (2.6 , 5.5)** | **3488.8 (2262.5 , 4798.5)** | **17887734 (12867706 , 23131993)** | **10.9 (7.9 , 13.9)** | **3777.2 (2692.6 , 4943.3)** | **8.3 (-6.5 , 28.8)** |
| **Afghanistan** | **301207 (151609 , 483585)** | **2.6 (1.3 , 4.1)** | **3923.7 (1946.8 , 6345.4)** | **791434 (500585 , 1153716)** | **4.6 (3 , 6.5)** | **5098.6 (3230.9 , 7244.7)** | **29.9 (-3.7 , 93.4)** |
| **Algeria** | **463576 (290907 , 676798)** | **4.8 (3.1 , 6.8)** | **3481.4 (2158.9 , 5095.7)** | **1177016 (829801 , 1579462)** | **11.6 (8.3 , 14.9)** | **3339.2 (2312.7 , 4478.5)** | **-4.1 (-26.1 , 28.9)** |
| **Bahrain** | **12145 (8470 , 16025)** | **9.8 (6.7 , 12.6)** | **5453.5 (3688.7 , 7355.3)** | **48979 (35007 , 63284)** | **17.2 (12.9 , 20.9)** | **4297.4 (2997.1 , 5612.6)** | **-21.2 (-35 , -3.1)** |
| **Egypt** | **1538733 (1006603 , 2115979)** | **5.4 (3.5 , 7.4)** | **4648.2 (2981.3 , 6435.6)** | **4217365 (2842298 , 5775492)** | **16 (11.3 , 20.3)** | **5929.6 (3973.1 , 8107.8)** | **27.6 (0.4 , 65.3)** |
| **Iran** | **710771 (443039 , 990856)** | **2.8 (1.8 , 4)** | **2419.6 (1497.6 , 3414.7)** | **1989457 (1441100 , 2561406)** | **10.1 (7.3 , 13)** | **2580.9 (1845.1 , 3337.3)** | **6.7 (-9.5 , 35.6)** |
| **Iraq** | **473081 (310127 , 644487)** | **6 (4 , 8.1)** | **5512.6 (3548.1 , 7546.6)** | **1236797 (841166 , 1672506)** | **12 (8.4 , 15.5)** | **4793.1 (3232.7 , 6505.3)** | **-13.1 (-30.6 , 9.3)** |
| **Jordan** | **67907 (46470 , 89035)** | **6.6 (4.5 , 8.6)** | **4595.7 (3077 , 6088.9)** | **262618 (190314 , 340017)** | **12.3 (9.1 , 15.1)** | **3701.6 (2637 , 4819.4)** | **-19.5 (-32.3 , -1)** |
| **Kuwait** | **30320 (22239 , 37964)** | **7.5 (5.5 , 9.4)** | **3581.5 (2514.8 , 4602.4)** | **108657 (80242 , 137859)** | **14.3 (11 , 17.3)** | **3156.1 (2248.5 , 4046.2)** | **-11.9 (-23.1 , 2.5)** |
| **Lebanon** | **84015 (52355 , 120019)** | **7 (4.4 , 9.7)** | **3499.4 (2176.7 , 5023.4)** | **181960 (122434 , 242468)** | **13.5 (9.2 , 17.5)** | **3488.6 (2344.6 , 4641.2)** | **-0.3 (-18.8 , 25.2)** |
| **Libya** | **66382 (44692 , 90778)** | **4.9 (3.3 , 6.6)** | **3132.2 (2088.8 , 4318.2)** | **225388 (156923 , 295467)** | **13.2 (9.5 , 16.7)** | **3921 (2681.3 , 5191.8)** | **25.2 (0.9 , 59.3)** |
| **Morocco** | **448994 (276690 , 651327)** | **4 (2.5 , 5.8)** | **2978.6 (1810.2 , 4339.5)** | **1296664 (840201 , 1804919)** | **12.8 (8.6 , 17.2)** | **3930.3 (2543 , 5491.5)** | **32 (2.7 , 77.5)** |
| **Oman** | **26630 (15381 , 39952)** | **4.3 (2.6 , 6.1)** | **3177.5 (1788.5 , 4868.1)** | **93026 (67425 , 119734)** | **10.9 (7.9 , 13.8)** | **4401.7 (3087.8 , 5712.6)** | **38.5 (2 , 109.5)** |
| **Palestine** | **31664 (18278 , 48533)** | **4.1 (2.5 , 6.1)** | **3419.1 (1948 , 5287.7)** | **95305 (65963 , 127230)** | **9.6 (6.5 , 12.8)** | **3647.4 (2444.6 , 4973.1)** | **6.7 (-16.4 , 43.7)** |
| **Qatar** | **8164 (5638 , 10617)** | **8 (5.6 , 10.2)** | **5514.9 (3644.2 , 7352.2)** | **57395 (42382 , 74978)** | **12.8 (10.1 , 15.2)** | **4904.9 (3498.2 , 6432.2)** | **-11.1 (-28.2 , 11.9)** |
| **Saudi Arabia** | **250956 (161135 , 353937)** | **4.6 (3.1 , 6.5)** | **3486 (2198.8 , 4998.6)** | **1183820 (845229 , 1535555)** | **14.2 (10.7 , 17.3)** | **4771.5 (3389.2 , 6141.8)** | **36.9 (4.1 , 86.8)** |
| **Sudan** | **290923 (153727 , 456906)** | **1.6 (0.8 , 2.4)** | **2760.8 (1445 , 4319.6)** | **839037 (541166 , 1189315)** | **6.6 (4.3 , 9.2)** | **3862.4 (2483.1 , 5500)** | **39.9 (4.8 , 116.8)** |
| **Syrian Arab Republic** | **254759 (152914 , 370622)** | **5.3 (3.2 , 7.6)** | **4102.1 (2441.4 , 6046)** | **525266 (332188 , 746285)** | **13.4 (8.8 , 18.1)** | **3994.5 (2515.4 , 5694.3)** | **-2.6 (-26.4 , 35)** |
| **Tunisia** | **131462 (84112 , 187399)** | **4.9 (3.2 , 7)** | **2450.4 (1548 , 3540.6)** | **374208 (244986 , 532923)** | **13 (8.8 , 17.3)** | **2916.8 (1907.4 , 4177.2)** | **19 (-7.6 , 58.1)** |
| **Turkey** | **1332294 (858478 , 1829630)** | **5.7 (3.7 , 7.7)** | **3470.2 (2224.3 , 4783.7)** | **2381067 (1609701 , 3206363)** | **12.1 (8.4 , 15.7)** | **2662.6 (1804 , 3599.4)** | **-23.3 (-37.6 , -5.4)** |
| **United Arab Emirates** | **38779 (26868 , 52806)** | **8.9 (6.2 , 11.7)** | **5877.8 (4059.1 , 7909.4)** | **373220 (272002 , 487534)** | **17.4 (13.6 , 21)** | **5732.9 (4111.7 , 7383.3)** | **-2.5 (-21.7 , 26.1)** |
| **Yemen** | **106305 (42181 , 189801)** | **1 (0.4 , 1.7)** | **1839.9 (730.4 , 3329.8)** | **410881 (228751 , 650581)** | **3.5 (2 , 5.2)** | **2595.6 (1422.7 , 4079.9)** | **41.1 (-1.6 , 137.9)** |

ASRs: Age-standardized rates; PAF: Population Attributable Fraction; UI: Uncertainty interval
